# Association between heavy metal levels and acute ischemic stroke

**DOI:** 10.1186/s12929-018-0446-0

**Published:** 2018-05-25

**Authors:** Ching-Huang Lin, Yi-Ting Hsu, Cheng-Chung Yen, Hsin-Hung Chen, Ching-Jiunn Tseng, Yuk-Keung Lo, Julie Y. H. Chan

**Affiliations:** 1 Department of Biological Sciences, National Sun Yet-Sen University, Kaohsiung, Taiwan; 2 Section of Neurology, Kaohsiung Veterans General Hospital, Kaohsiung, Taiwan; 3 Department of Medical Education and Research, Kaohsiung Veterans General Hospital, Kaohsiung, Taiwan; 4 Department of Physical Therapy, Shu-Zen Junior College of Medicine and Management, Kaohsiung, Taiwan; 5 Institute for Translational Research in Biomedicine, Kaohsiung Chang Gung Memorial Hospital, Kaohsiung, 83301 Taiwan

**Keywords:** Stroke, Acute ischemic stroke, Heavy metal, Lead, Mercury, Arsenic, Cadmium

## Abstract

**Background:**

Few studies have examined the relationship between the amounts of heavy metal and stroke incidence. The aim of this study was to explore the relationship between levels of heavy metals, including Pb, Hg, As, and Cd, in patients with acute ischemic stroke (AIS).

**Methods:**

We selected patients with first-ever AIS onset within 1 week as our study group. Healthy controls were participants without a history of stroke or chronic disease, except hypertension. The serum levels of Pb, Hg, As, and Cd in participants in the experimental and control groups were determined. All participants received a 1-g infusion of edetate calcium disodium (EDTA). Urine specimens were collected for 24 h after EDTA infusion and measured for heavy metal levels.

**Results:**

In total, 33 patients with AIS and 39 healthy controls were enrolled in this study. The major findings were as follows: (1) The stroke group had a significantly lower level of serum Hg (6.4 ± 4.3 μg/L vs. 9.8 ± 7.0 μg/L, *P* = 0.032, OR = 0.90, 95% CI = 0.81–0.99) and a lower level of urine Hg (0.7 ± 0.7 μg/L vs. 1.2 ± 0.6 μg/L, *P* = 0.006, OR = 0.27, 95% CI = 0.11–0.68) than the control group. (2) No significant difference in serum Pb (S-Pb), As (S-As), and Cd (S-Cd) levels and urine Pb (U-Pb), As (U-As) and Cd (U-Cd) levels was observed in either group.

**Conclusions:**

Our study found low levels of serum and urine Hg in first-ever patients with AIS, providing new evidence of dysregulated heavy metals in patients with AIS.

**Electronic supplementary material:**

The online version of this article (10.1186/s12929-018-0446-0) contains supplementary material, which is available to authorized users.

## Background

Cerebrovascular disease (CVD) is among the three commonest causes of deaths worldwide and is the commonest cause of disability [1–3]. Modifiable risk factors are high blood pressure, dyslipidemia, diabetes, smoking, alcohol consumption, obesity, and other metabolic syndromes [4]. Because of industrial development and environmental pollution, the contribution of environmental toxins and heavy metals to diseases such as stroke warrants thorough investigation.

Studies have suggested that Pb severely damages the endothelium in the brain vasculature and induces cerebral microvascular dysfunction with subsequent changes in the cerebral blood flow [5–9]. Studies have suggested that long-term Pb exposure, measured using the body Pb store, increases the risk of intracranial carotid atherosclerosis and is related to CVD or stroke [10] Hence, Pb is likely involved in cerebral atherosclerosis pathogenesis and may cause cellular toxicity and pathological damage and may be related to CVDs [5–7, 11].

Hg may predispose individuals to atherosclerotic disease by increasing free radical production, oxidative stress, thrombosis, and vascular inflammation [12]. Hg toxicity includes hypertension-increased carotid intima-media thickness, carotid artery obstruction, and cerebrovascular accidents [13]. Although fish consumption protects against CVD because of the effect of omega-3 fatty acids, eicosapentaenoic acid (EPA), and docosahexaenoic acid (DHA), it also exposes individuals to Hg, which can increase the likelihood of developing CVDs [13].

A study reported that As harms the central and peripheral nervous systems as well as the heart and blood vessels [14]. Epidemiological studies have shown that chronic As poisoning is associated with various CVDs, including carotid atherosclerosis, impaired microcirculation, hypertension, coronary artery disease, and cerebral infarction (stroke) [15].

Cd has been demonstrated to induce vascular disorders such as atherosclerosis in animals [16], promote arterial vessel wall proliferation, and influence the synthesis of proteoglycan and fibrinolysis in human studies [5, 16, 17] and has been associated with significantly increased stroke and heart failure prevalence in epidemiological studies [17, 18].

Heavy metal toxicity can be identified in diseases that include thrombosis, hypertension, coronary heart disease, myocardial infarction, cardiac arrhythmias, increased carotid intima-media thickness, carotid artery obstruction, generalized atherosclerosis, and cerebrovascular accidents [13]. Evidence on the association of heavy metals with the risk of stroke, however, remains inconclusive. Previous studies have indicated that long-term Pb exposure, measured using the body Pb store, could increase the risk of intracranial carotid atherosclerosis and may be related to CVD or stroke [10]. Many basic mechanisms allow heavy metals to increase or reduce the risk of stroke, but they have not been adequately studied. The aim of this study was to explore heavy metal levels in patients with acute ischemic stroke (AIS).

## Methods

### Study design and participants

Patients who were admitted to the Department of Neurology of Kaohsiung Veterans General Hospital with a first-ever diagnosis of AIS within 3–7 days after stroke onset were enrolled in this study during 2012–2013. This study was approved by the Institutional Review Board of Kaohsiung Veterans General Hospital, and the requirement for written informed consent from the patients was waived. The Medical Ethics Committee of the hospital approved the study protocol (VGHKS 11-CT5–04).

The healthy controls were volunteers without stroke and chronic CVDs, except hypertension. Informed consent was obtained from all participants, legally authorized representatives, or next of kin. The exclusion criteria were as follows: the patient was sensitive to edetate calcium disodium (EDTA), had intraparenchymal hemorrhage (ICH) verified through brain imaging, had a stroke etiology that was considered related to tumor or cardiac embolism, required immediate surgical intervention, was in critical condition requiring intensive care in the intensive care unit, was participating in another interventional clinical trial, or was pregnant, had undergone parturition within the preceding 30 days, or was actively lactating.

### Study procedures (Fig. 1)

All enrolled patients and healthy controls received the following routine medical examinations: electrocardiogram, complete blood count, blood glucose, electrolyte and lipid profiling, and neuroimaging before measurement of heavy metals. Neuroimaging included computed tomography and magnetic resonance imaging to detect evidence of AIS. A questionnaire, which included questions on baseline characteristics, residence, eating habits, and stroke risk factors, was completed by each enrolled subject. The definitions of stroke risk factors were defined as bellows: Hypertension was defined as subject had history of hypertension and/or took hypotensive agent in medical chart and/or blood pressure≧140/90 mmHg for 2 occasions during hospitalization or visit. Diabetes was defined as subject has history of diabetes, and or took hypoglycemic agent in medical chart and/or fasting blood sugar level ≧126 mg/dL on two separate tests during hospitalization or visit. Hyperlipidemia was defined as subject had history of hypercholesterolemia and/or hypertriglyceridemia and/or took lower-lipid agent in medical chart and/or fasting blood test showed total cholesterol is above 200 mg/dl, and/or LDL > 100 mg/dl and/or TG > 150 mg/dl. Smoking was defined as subject is current smokers who reported cigarette use by himself/herself at the time of survey regardless of levels of smoking, and/or subject abstained from smoking less than 2 years. Alcohol consumption was defined as subject reported alcohol consumption at least one time/week regardless of amount by himself/herself at the time of survey and/or subject abstained from alcohol consumption less than 2 years. The frequency of fish consumption was surveyed for each subject by using a questionnaire to divide into two groups: marine fish group and freshwater fish group. The marine fish group was defined as the subject consumed marine fish:≧3 time/week, the amount of marine fish consumption was above the amount of freshwater fish consumption. The freshwater fish was defined as the subject consumed freshwater fish:≧3 time/week, the amount of freshwater fish consumption was above the amount of marine fish consumption.

**Fig. 1:**
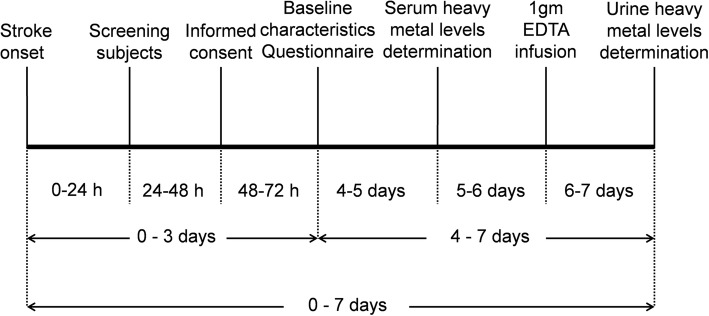
Flow chart of the study

### Measurements of heavy metal levels

The serum specimens of Pb, Hg, As, and Cd were collected before infusion of the heavy metal chelating agent (EDTA). Urine specimens were collected for 24 h after EDTA infusion within post-stroke one week. The 24 h’s urine sample was collected for 10 ml after shaking evenly. The 10 ml serum and urine samples were sent to the Department of Clinical Toxicology in Taipei Veterans General Hospital, a reliable and qualified lab center for testing heavy metals in Taiwan, by immediate delivery service. The heavy metals may accumulate in relatively non-exchangeable pools within specific tissues such as brain, liver and kidneys. In some conditions, it cannot show the total burden of heavy metals by static samples of blood and urine. The acceptable method for confirmation of total heavy metals burden is used a chelating agent to bind heavy metals and redistribute into the blood, and be eliminated in the urine.

EDTA is a chelating agent, also known as sodium calcium edetate (sodium calcium EDTA) [19]. The pharmacologic effects of EDTA are due to chelate formation with divalent and trivalent metals, making it to move from the blood stream to the urine [19]. EDTA-based chelation therapy was used in patients with CVD because calcium chelation may stabilize or reduce atherosclerotic plaques containing calcium [20]. Other putative beneficial effects of EDTA in CVD include free radical scavenging, cell membrane stabilization, arterial dilatation because of reduced calcium channel activity, arterial wall elasticity improvement, increased nitric oxide production, and a reduction in Pb and Cd levels [20–24]. EDTA was injected by intravenous (IV) infusion with an EDTA dose of 1 g/250 mL of 0.9% sodium chloride or 5% dextrose for 1–2 h [19].

Heavy metal levels were quantified using an inductively coupled plasma–mass spectrometer (ICP–MS) at the Department of Clinical Toxicology in Taipei Veterans General Hospital. The ICP–MS is a type of mass spectrometer that can ascertain the concentration of heavy metals and several nonmetals at concentrations as low as one part in 1015 (part per quadrillion, ppq) in no interfered low-background isotopes [25, 26].

The ICP–MS identifies elements with atomic masses ranging from 7 to 250 (Li to U) and sometimes higher. In contrast to atomic absorption spectroscopy, which can only measure one element at a time, ICP–MS can simultaneously scan for all elements. Its rapid scanning, large dynamic range, and large mass range are suitable for measuring multiple unknown concentrations and isotope ratios in samples that have had minimal preparation, for example, seawater, urine, and digested whole rock samples [27]. ICP–MS is used clinically, in cases such as suspected heavy metal poisoning, metabolic concerns, and hepatological problems, and can analyze serum, urine, and white and red blood cells [26].

In determining serum As, Pb and Cd amounts, 0.3 ml blood sample was mixed with 0.5 ml EDTA, 0.5 ml Cysteine and 3.5 ml mixed solution before quantifying serum As, Pb and Cd levels. In determining serum and urine Hg amounts, 0.3cm^3^ serum and urine sample were mixed with 0.5 ml EDTA, 0.5 ml Cysteine and 3.5 ml HCL in each before quantifying serum and urine Hg levels. In determining the levels of urine As, Pb and Cd, 1 ml urine sample was mixed with 0.5 ml EDTA, 0.5 ml Cysteine and 4 ml HNO3 before quantifying urine As, Pb and Cd levels.

### Statistical analysis

Statistical analysis was performed using SPSS v. 20 (IBM SPSS Statistics 20, USA). An independent *t* test was used to compare the heavy metal levels between the AIS and control groups. Pearson’s chi-squared test was used to compare the differences by sex, diabetes status, hypertension, hyperlipidemia, smoking habit, alcohol consumption, marine fish consumption, freshwater fish consumption, and seafood consumption of the two groups.

The associations between demographic characteristics and each heavy metal concentration were analyzed using unadjusted and adjusted logistic regression models. The results are presented as the significance (*P* value), odds ratio (OR), and corresponding 95% confidence interval (CI).

## Results

### Baseline characteristics of the stroke group and the control group

Thirty-three patients with first-ever AIS and 39 healthy controls were enrolled during the study period. The mean age was 57.7 ± 10.5 among patients with AIS compared with 58.9 ± 7.6 among the healthy controls (Table 1). No statistically significant difference was observed in age between the two groups. The stroke group had significantly higher levels of body mass index, systolic blood pressure(SBP), diastolic blood pressure(DBP), fasting blood sugar, HbA1c, triglyceride(TG), and higher rates of hypertension, diabetes, dyslipidemia, smoking, and alcohol consumption than the control group.

**Table 1:** Baseline demographic characteristics of stroke and control groups

Variables	Stroke (*n* = 33)	Control (*n* = 39)	*P*
Age (y/o, *mean ± SD*)	57.7 ± 10.5	58.9 ± 7.6	0.601
BMI (*mean ± SD*)	25.7 ± 3.8	23.3 ± 2.2	0.002
Gender (male, %)	27 (81.8%)	20 (51.3%)	0.007
Hypertension (*n*, %)	27 (81.8%)	13 (33.3%)	< 0.001
SBP (mmHg, *mean ± SD*)	125.4 ± 17.5	106.2 ± 11.9	< 0.001
DBP (mmHg, *mean ± SD*)	80.3 ± 11.5	68.9 ± 9.9	< 0.001
Diabetes (*n*, %)	13 (39.4%)	1 (2.6%)	< 0.001
FBG (mg/dL, *mean ± SD*)	118.6 ± 40.9	89.6 ± 8.5	< 0.001
HbA1c (%, *mean ± SD*)	6.9 ± 1.8	5.8 ± 0.3	0.004
Hyperlipidemia (*n*, %)	30 (90.9%)	21 (53.8%)	0.001
TG (mg/dL, *mean ± SD*)	191.9 ± 122.9	128.7 ± 90.9	0.015
CHOL (mg/dL, *mean ± SD*)	203.9 ± 30.3	190.7 ± 29.0	0.064
Smokes (*n*, %)	17 (51.5%)	3 (7.7%)	< 0.001
Alcohol consumption (*n*, %)	9 (27.3%)	0 (0.0%)	0.005

*BMI* body mass index*, SBP* systolic blood pressure*, DBP* diastolic blood pressure*, FBG* fasting blood sugar*, TG* triglyceremia*, CHOL* cholesterol

### The association between serum heavy metal levels and body mass index, hypertension, diabetes, dyslipidemia (all patients)

There were no significant associations between serum heavy metals (serum Pb (S-Pb), serum Hg(S-Hg), serum As (S-As), and serum Cd (S-Cd)) and other stroke risk factors (body mass index(BMI), HbA1c, fasting blood sugar(FBG), Triglyceride(TG), total cholesterol(Chol), SBP (systolic blood pressure) and DBP (diastolic blood pressure) in all subjects (stroke patient and normal control) (Table 2).

**Table 2:** The association between serum heavy metal levels and body mass index, hypertension, diabetes, dyslipidemia (all patients)

Variables	S-Pb (μg/L)		S-Hg (μg/L)		S-As (μg/L)		S-Cd (μg/L)	
	Correlation Coefficient^a^	*p*-value	Correlation Coefficient^a^	*p*-value	Correlation Coefficient^a^	*p*-value	Correlation Coefficient^a^	*p*-value
BMI	−0.066	0.582	0.180	0.139	0.072	0.550	−0.032	0.793
HbA1c	−0.118	0.348	−0.207	0.106	−0.121	0.338	−0.180	0.152
FBG	−0.096	0.430	−0.095	0.446	−0.021	0.860	−0.110	0.363
TG	−0.076	0.530	0.065	0.596	−0.065	0.588	0.043	0.721
CHOL	−0.041	0.733	−0.097	0.428	−0.148	0.216	0.019	0.874
SBP	−0.055	0.682	−0.050	0.717	−0.028	0.835	−0.007	0.959
DBP	0.009	0.944	0.211	0.125	0.048	0.721	0.088	0.516

^a^The correlation coefficient and p-value were estimated by Pearson’s correlation coefficient

*BMI* body mass index*, SBP* systolic blood pressure*, DBP* diastolic blood pressure*, FBG* fasting blood sugar*, TG* triglyceremia*, CHOL* cholesterol

### The levels of heavy metal levels in the stroke and control groups & the significance of heavy metals in stroke prediction

No significant difference in serum Pb (S-Pb), As (S-As), and Cd (S-Cd) levels and urine Pb (U-Pb), As (U-As) and Cd (U-Cd) levels was observed in either group (Table 3). The stroke group had significantly lower levels of serum Hg (S-Hg) (6.4 ± 4.3 *m*g/L vs. 9.8 ± 7.0 μg/L, *P* = 0.032) and significantly lower levels of urine Hg (U-Hg) compared with the control group (0.7 ± 0.7 μg/L vs. 1.2 ± 0.6 μg/L, *P* = 0.006). Both groups had similar seafood consumption habits and marine fish consumption habits. However, patients with stroke had a significantly higher rate of freshwater fish consumption compared with the healthy controls (90.9% vs. 61.5%, *P* = 0.008).

**Table 3:** Univariate logistic regression of patients with stroke and healthy controls using serum or urine heavy metal levels and fish consumption as predictors

Variables	Stroke (*n* = 33)	Control (*n* = 39)	*Odds ratio*	*95% CI*	*P*
	Mean ± SD	Mean ± SD			
S-Pb (μg/L)	21.5 ± 8.4	24.6 ± 9.8	0.97	0.91–1.02	0.162
S-Hg (μg/L)	6.4 ± 4.3	9.8 ± 7.0	0.90	0.81–0.99	0.032
S-As (μg/L)	5.6 ± 5.5	7.5 ± 4.7	0.92	0.82–1.03	0.134
S-Cd (μg/L)	0.8 ± 0.5	0.7 ± 0.3	1.50	0.42–5.42	0.534
U-Pb (μg/L)	11.3 ± 7.4	10.7 ± 6.8	1.01	0.95–1.08	0.754
U-Hg (μg/L)	0.7 ± 0.7	1.2 ± 0.6	0.27	0.11–0.68	0.006
U-As (μg/L)	53.2 ± 63.2	94.7 ± 138.0	1.00	0.99–1.00	0.178
U-Cd (μg/L)	2.1 ± 2.2	1.2 ± 1.0	1.47	0.98–2.19	0.061
Marine Fish (*n*, %)	28 (84.8%)	29 (74.4%)	1.93	0.59–6.36	0.279
Freshwater Fish (*n*, %)	30 (90.9%)	24 (61.5%)	6.25	1.62–24.13	0.008
Seafood (*n*, %)	30 (90.9%)	31 (79.5%)	2.58	0.63–10.66	0.190

S*-Pb* serum lead, *S-Hg* serum mercury, *S-As* serum arsenic, *S-Cd* serum cadmium, *U-Pb* urine lead, *U-Hg* urine mercury, *U-As* urine arsenic, *U-Cd* urine cadmium, *CI* confidence interval

The results of univariate logistic regression using serum and urine heavy metal levels as stroke predictors in patients with stroke versus the healthy controls are shown in Table 3. The S-Hg level in patients with AIS was significantly lower than that in the healthy controls (6.4 ± 4.3 μg/L vs. 9.8 ± 7.0 μg/L, *P* = 0.032, OR = 0.90, 95% CI = 0.81–0.99). The level of U-Hg in patients with AIS was significantly lower than that of the healthy controls (0.7 ± 0.7 μg/L vs. 1.2 ± 0.6 μg/L, *P* = 0.006, OR = 0.27, 95% CI = 0.11–0.68). Statistically significant “protective” effects of S-Hg and U-Hg exposure were observed only in patients with AIS (OR = 0.90 and 0.27, respectively).

The results of stepwise multiple logistic regression using S-Hg and U-Hg levels as stroke predictors in patients with stroke compared with the healthy controls after controlling for key demographic characteristics are shown in Tables 4 and 5. Table 4 shows that S-Hg has an independently significant inverse association with stroke prediction (stepwise multiple logistic regression, *P* = 0.004, OR = 0.73, 95% CI = 0.59–0.91). Table 5 shows that U-Hg has an independently significant inverse association with stroke prediction (stepwise multiple logistic regression, *P* = 0.006, OR = 0.02, 95% CI = 0.002–0.34). Additionally, both freshwater fish consumption and hyperlipidemia have independently positive associations with stroke prediction (Tables 4 and 5).

**Table 4:** Stepwise multiple logistic regression of patients with stroke and healthy controls using serum Hg level as a stroke predictor after controlling for key demographic characteristics

Variables	*Odds ratio*	*95% CI*	*P*
Freshwater fish	30.71	1.92–490.63	0.015
Hyperlipidemia	44.09	3.16–614.96	0.005
S-Hg	0.73	0.59–0.91	0.004

**Table 5:** Stepwise multiple logistic regression of patients with stroke and healthy controls using urine Hg level as a stroke predictor after controlling for key demographic characteristics

Variables	*Odds ratio*	*95% CI*	*P*
Freshwater fish	45.34	2.42–851.38	0.011
Hyperlipidemia	55.14	3.41–892.92	0.005
U-Hg	0.02	0.002–0.34	0.006

### Difference in serum and urine heavy metal levels for marine and freshwater fish consumption

All enrolled participants were surveyed to evaluate their marine and freshwater fish consumptions habits. Table 6 shows that no significant differences in S-Pb, S-Cd, S-As, U-Pb, U-Hg, U-Cd, and U-As levels were observed in participants who had a marine fish consumption habit compared with those who did not (Table 5). However, participants with a marine fish consumption habit had significantly higher S-Hg levels compared with those who did not (9.4 ± 6.4 μg/L vs. 4.0 ± 2.4 μg/L, *P* < 0.001). Table 7 reveals that no significant differences in S-Pb, S-Hg, S-Cd, S-As, U-Pb, U-Hg, U-Cd, and U-As were observed in participants who had a freshwater fish consumption habit compared with those who did not.

**Table 6:** Comparison of serum and urine heavy metal levels for participants who consume marine fish and those who do not

Variables	Marine fish Consumption	Marine fish Non-consumption	*P*
	Mean ± SD	Mean ± SD	
S-Pb (μg/L)	23.3 ± 9.6	22.7 ± 8.0	0.838
S-Hg (μg/L)	9.4 ± 6.4	4.0 ± 2.4	< 0.001
S-As (μg/L)	6.9 ± 5.2	5.7 ± 5.1	0.432
S-Cd (μg/L)	0.8 ± 0.4	0.6 ± 0.3	0.081
U-Pb (μg/L)	11.3 ± 7.3	9.7 ± 6.0	0.419
U-Hg (μg/L)	1.1 ± 0.7	0.7 ± 0.5	0.098
U-As (μg/L)	86.0 ± 122.9	36.5 ± 21.6	0.127
U-Cd (μg/L)	1.7 ± 1.8	1.2 ± 1.0	0.345

S*-Pb* serum lead, *S-Hg* serum mercury, *S-As* serum arsenic, *S-Cd* serum cadmium, *U-Pb* urine lead, *U-Hg* urine mercury, *U-As* urine arsenic, *U-Cd* urine cadmium, *CI* confidence interval

**Table 7:** Comparison of serum and urine heavy metal levels for participants who consume freshwater fish and those who do not

Variables	Freshwater fish Consumption	Freshwater fish Non-consumption	*P*
	Mean ± SD	Mean ± SD	
S-Pb (μg/L)	22.9 ± 9.5	23.9 ± 8.4	0.695
S-Hg (μg/L)	8.4 ± 5.9	7.8 ± 7.2	0.725
S-As (μg/L)	6.4 ± 4.7	7.2 ± 6.4	0.585
S-Cd (μg/L)	0.8 ± 0.4	0.7 ± 0.3	0.355
U-Pb (μg/L)	11.1 ± 7.2	10.6 ± 6.8	0.823
U-Hg (μg/L)	1.0 ± 0.7	1.0 ± 0.7	0.782
U-As (μg/L)	75.8 ± 109.6	75.2 ± 120.2	0.983
U-Cd (μg/L)	1.7 ± 1.8	1.3 ± 1.2	0.343

S*-Pb* serum lead, *S-Hg* serum mercury, *S-As* serum arsenic, *S-Cd* serum cadmium, *U-Pb* urine lead, *U-Hg* urine mercury, *U-As* urine arsenic, *U-Cd* urine cadmium, *CI* confidence interval

## Discussion

Evidence increasingly indicates that exposure to toxicological heavy metals, such as Pb, Hg, As, and Cd, is linked to cardiovascular risks as well as stroke [5, 6, 11, 28]. Several epidemiological studies have demonstrated that the incidence of stroke was associated with higher Pb levels [28, 29]. Additionally, Pb exposure as a contributing factor to the incidence of CVDs, including stroke, has been reported [30, 31]. In the present study, we found no significant difference in S-Pb levels in patients with AIS compared with the control group. Both groups had a higher U-Pb level than the normal level (U-Pb levels should be less than 7.5 μg/g; see Additional file 1). Our study was the first to evaluate simultaneously the blood and urine amounts of heavy metals in first-ever patients with AIS with stroke onset within 1 week.

Planchart et al. reviews 36 epidemiological studies and 13 studies leveraging model systems to evaluate the possible associations between heavy metal exposure (Pb, Cd and Hg) and metabolic syndrome or comorbid condition, but the results remain conflicting [32]. In our study, there were no significant associations between serum heavy metals and other stroke risk factors (Table 2).

Studies have shown that Hg exposure had positive and negative associations with CVD. Positive associations between the levels of Hg in the hair or nails and increased cardiovascular risk have been reported in epidemiological studies [33, 34]. One study reported that mercury in the blood may be associated with increased risk of hypertension and myocardial infarction or angina [12], but no association with stroke [12]. Another study among US health professionals found that no association existed between Hg level in nail and risk of coronary heart disease, stroke or cardiovascular disease [35]. By contrast, protective associations have been found between S-Hg levels and the risk of acute myocardial infarction [36] but not stroke [37] in prospective case–control studies from northern Sweden. In a cohort study of women with previously determined S-Hg in Gothenburg, Sweden, higher S-Hg levels predicted low mortality and a low risk of fatal AMI but had no predictive value for nonfatal myocardial infarction or stroke [38]. In our study, both S-Hg and U-Hg had a significantly protective effect against stroke after adjustment for sex, age, hypertension, and other key demographic characteristics. Our study is the first to document the negative associations between the levels of S-Hg and U-Hg and stroke.

Several studies have shown that elevated Hg levels were positively associated with fish consumption [39, 40]. Therefore, we investigated fish consumption habits by using a questionnaire. We found a statistically significant association between S-Hg and marine fish consumption (Table 6). However, no statistical significance was observed between S-Hg and freshwater fish consumption (Table 7). Previous studies showed that fish consumption was associated with decreased cardiovascular risk [41]. The nutrients in fish, particularly the long-chain n-3 polyunsaturated fatty acids (PUFAs), lead to a reduction in cardiovascular risk. However, fish are rich in Hg, particularly marine fish, which causes oxidative stress and predisposes individuals to atherosclerotic disease. Hg could counteract the beneficial effects of the PUFAs, EPA, and DHA in fish. However, in this study, high S-Hg and U-Hg levels protected against stroke. The counteractive effects of Hg and fish nutrients (PUFAs, EPA, and DHA) require comprehensive study.

The geometric mean of total blood mercury level was 5.07 μg/L in Korean males and 3.59 μg/L in Korean females from Korea National Health and Nutrition Examination Survey (KNHANES, 2008–2012) [42], 0.23 μg/L in the German Environmental Survey IV (2003–2008) [43], 0.70 μg/L based on the 2011–2012 NHANES in USA [44] and 3.13 μg/L in Bogota [45]. In Taiwan, the geometric mean of total blood mercury levels was 9.1 μg/L, 89 % of the maternal blood mercury concentrations exceeded the US National Research Council recommended value of 5.8 μg/L [46]. The Asian group had the highest blood mercury level among USA populations [44]. In our study, the mean of total blood mercury level is 6.4 ± 4.3 μg/L in stroke group and 9.8 ± 7.0 μg/L in control group. The blood mercury level was higher among Taiwanese and Asians had been reported in previous studies and became a more concerned issue.

In an epidemiologic study, highest As exposure was linked to CVD, including coronary heart disease, peripheral arterial disease, and stroke [47]. In several studies, Cd has been shown to induce vascular disorders, such as atherosclerosis, in animals. Environmental exposure to Cd has been associated with a significant increase in stroke prevalence [5, 17, 18]. In the present study, S-As, U-As, S-Cd and U-Cd levels exhibited no significant difference in patients with AIS compared with the control group (Table 3).

### Limitations

The present study has some limitations. First, our sample size was relatively small and the study was performed at a single institution. Thus, the results cannot be generalized to general population. Second, some covariates such as alcohol consumption, smoking habit and fish consumption habit were self-reported, and thus recall bias is inevitable. Third, a single blood/urine sample does not reflect the total body burden despite chelating agent was performed. There are no standard reference data of urinary heavy metal levels. Therefore, the associations found in this study need to be further investigated in future studies.

## Conclusion

Our study was the first to evaluate the amount of heavy metals in the blood and urine of patients with stroke in the acute phase. The findings of this study indicated that patients with stroke had lower levels of U-Hg and S-Hg, providing new evidence of the potential association of dysregulated heavy metals in patients with AIS. Furthermore, negative associations between U-Hg and S-Hg levels and stroke were revealed. Additionally, marine fish consumption may be significantly associated with S-Hg levels. However, because our participant numbers were limited, future studies must elucidate the roles of the identified heavy metals in the prevalence and prognosis of AIS and other CVDs.

## Additional file

Additional file 1: Questionnaire. (DOC 39 kb)

## Abbreviations

AIS: Acute ischemic stroke
CVD: Cerebrovascular disease
DHA: Docosahexaenoic acid
EDTA: Edetate calcium disodium
ICH: Intraparenchymal hemorrhage
ICP–MS: Inductively coupled plasma–mass spectrometer
PUFAs: Polyunsaturated fatty acids
