# Supplementary material for: Association between heavy metal levels and acute ischemic stroke
Source: J Biomed Sci. 2018 May 25;25:49. doi: 10.1186/s12929-018-0446-0 (PMC5970463; doi:10.1186/s12929-018-0446-0)
Supplement: Supplementary file 1 — Questionnaire. (DOC 39 kb) [file 12929_2018_446_MOESM1_ESM.doc]

**Appendix**

***Questionnaire***

**1**、General Data

| Name： | Medical record No.： | | Gender：□ male □ female | |
| --- | --- | --- | --- | --- |
| Birth day： (dd/mm/yyyy) | Height： cm | Weight： kgw | | Occupation： |
| Telephone No.: | Correspondence Address： | | | |
| Mobile No.： |

**2. Resident Place in Taiwan**：□ Southern □ Middle □ Northern □ Eastern

**3、Dietary Habit**：□ non-vegetarian、□ vegetarian (＞1yr)

(1). Time of dinner：between ____ pm and ___ pm

(2). Breakfast：□ no □ yes, at ___ am

(3). What kind of meat do you eat：
□ Beef □ Mutton □ Pork □ Chicken □ No (multi-select)
□ Beef □ Mutton □ Pork □ Chicken (most frequently eat )

(4). What kind of fish do you eat frequently：
□ Marine fish □ Freshwater fish (include fresh water) □ No

(5). Do you eat seafood other than fish? : □ yes □ no

(6). How many kinds of fruit do you eat every day? ＿＿kinds of fruit per day
(7). How many kinds of vegetable do you eat every day? ＿＿kinds of vegetable per day

(8). How much water do you drink every day? (include water, soup and drink)：

□ ＜1000 c.c. □ 1000~2000 c.c. □＞2000 c.c.

**4**、Risk factor of stroke (0：no、1：yes、2：unknown)

| □ Hypertension, Duration: ＿ year | □ Diabetes mellitus, Duration: ＿ year | □ Hyperlipidemia  　□ Hypercholesterolemia  □ Hypertriglyceridemia | □ Smoking  □ No or quitted ≧2 yrs  □ Yes:＿＿cigarettes/day  Duration: ＿ year |
| --- | --- | --- | --- |
| □ Alcoholism,  Duration: ＿ year | □ Heart disease,  What kind ________ |  |  |
| □ History of transient ischemic attack | □ History of stroke  □ Ischemic  □ Hemorrhagic | □ History of peripheral arterial occlusive disease |  |
|  |  |  | □ Hyperuricemia |
